# Work-related head injury and industry sectors in Finland: causes and circumstances

**DOI:** 10.1007/s00420-022-01950-9

**Published:** 2023-01-03

**Authors:** Aura Heimonen, Kari Nousiainen, Heikki Lassila, Ari Kaukiainen

**Affiliations:** 1 Faculty of Medicine, Department of Oral and Maxillofacial Diseases, University of Helsinki, PO BOX 41, 00014 Helsinki, Finland; 2 LocalTapiola General, LähiTapiola, 02010 Espoo, Finland; 3 Faculty of Medicine, Department of Public Health, University of Helsinki, PO BOX 20, 00014 Helsinki, Finland

**Keywords:** Construction industry, Violence, Health care, Head injury, Occupational accident, ESAW

## Abstract

**Objective:**

Despite the continuous development of occupational safety, the prevalence of work-related head injuries is excessive. To promote prevention, we conducted a study evaluating the risks and pathways that precede head injuries in different economic activity sectors.

**Methods:**

In Finland, more than 90% of employees are covered by inclusive statutory workers’ compensation. We obtained data on occupational head injuries in 2010–2017 from an insurance company database. The European Statistics on Accidents at Work (ESAW) variables represented the characteristics of the accidents and the injury. We analysed the risk factors, contributing events and injury mechanisms in 20 industry sectors, based on the Statistical Classification of Economic Activities in the European Community (NACE).

**Results:**

In the 32,898 cases, the most commonly affected area was the eyes (49.6%). The highest incidence of head injuries was in construction (15.7 per 1000 insurance years). Construction, manufacturing, and human health and social work activities stood out due to their distinctive ESAW category counts. ‘Working with hand-held tools’ [risk ratio (RR) 2.23, 95% confidence interval (CI) 2.14–2.32] in construction and ‘operating machines’ (RR 3.32, 95% CI 3.01–3.66) and ‘working with hand-held tools’ (1.99, 1.91–2.07) in manufacturing predicted head injury. The risk related to parameters of violence and threats in health and social work activities was nearly ninefold the risk of other sectors.

**Conclusion:**

The risks and pathways preceding head injuries varied considerably. The highest head injury rates were in construction and manufacturing. Violence emerged as a major risk factor in human health and social work activities.

## Introduction

An ‘accident at work’ or an ‘occupational accident’ is defined in the European Statistics on Accidents At Work (ESAW) methodology as a discrete occurrence during the course of work that leads to physical or mental harm and more than 3 days absence from work (EU 2013). In the EU in 2018, only 6% of the total of body parts injured in non-fatal occupational accidents concerned the head. However, of fatal injuries at work, 24% were head injuries (EU 2020), and head injury is a common cause of death and disability (Majdan et al. 2016; Toccalino et al. 2021). The economic and societal burden of serious forms of head injuries is significant (Majdan et al. 2016).

More knowledge is available on traumatic brain injury (TBI) than on other work-related head injuries. The risk of TBI is elevated in primary and construction industries, and in general among male workers, with falls being the most common mechanism of the injury (Chang et al. 2015). Commonly reported industries with a high prevalence of TBI include education and training, healthcare and social assistance, manufacturing and transportation (Toccalino et al. 2021). The most common type of work-related head injuries in the construction industry are wounds and superficial injuries (49%), followed by other specified injuries (21%) and TBI (11%) (Brolin et al. 2021). Eye injuries also occur and their risk is higher among males, among younger and less experienced workers, and in manual tasks (Martin-Prieto et al. 2020).

Finland, with a workforce of 2.5 million, has an inclusive social security system and high coverage of statutory workers’ compensation insurance and occupational health services (OHS) (OSF 2021a; Rantanen et al. 2017). A total of 135,500 accidents occurred at work in Finland in 2018 (OSF 2021b). In line with European statistics (EU 2020), the upper and lower extremities were the most common body parts injured, followed by the head, with eye injuries being the most common form of head injury (TVK 2020). In Finland, the number of the occupational head injuries remained annually constant in 2010–2017, i.e. 16,000 cases, when injuries during commuting were excluded.

Despite continuous attempts to improve occupational safety in industry sectors, too many work-related head injuries still occur. To promote their prevention, we conducted a study to evaluate the incidents that precede work-related head injuries in different economic activity sectors. We used the database of a major Finnish insurer, as the mandatory workers’ compensation statistics cover a high percentage of the workforce.

## Materials and methods

### Design

Our register-based descriptive, retrospective study evaluated the risks and pathways of the events preceding work-related head injuries in 20 industry sectors in Finland. The risk profiles of the different industry sectors were examined by counting the annual injury densities, i.e. the annual incidence of injuries causing head traumas per 1000 insurance years.

### Data sources

According to legislation, employer submits a notice of the occupational accident to insurance company including ESAW classification codes. We obtained data from the workers’ compensation database of LocalTapiola General Mutual Insurance Company, one of the three largest national insurers (market share of about 30%). All reported work-related injuries are recorded in the database. The cases were head injuries at work in 2010–2017, commuting and leisure-time injuries and verified occupational diseases excluded. The database includes information on ESAW variables, representing the conditions in which the work-related injuries occurred (EU 2013). ESAW also includes information on the body parts injured (such as the head, neck, back, torso and organs, arms and hands, legs and feet). The economic activity sectors were classified according to the Standard Industrial Classification (TOL) 2008 (OSF 2008), like the Statistical Classification of Economic Activities in the European Community (NACE) (EU 2008), with the exception of one additional code (X, Industry unknown).

### Variables

The following ESAW variables concerning body part(s) injured were included, with the corresponding code numbers: 11, head (caput), brain, and cranial nerves and vessels; 12, facial area; 13, eye(s); 14, ear(s); 15, teeth; 18, head, multiple sites affected, 19, head other parts not listed. Our study used the TOL code on economic sectors, with 20 fields of activity. In addition, we obtained the following data for each year: gender, date of birth and date of injury, working hours, the number of person years (person year = 1600 working hours) and the number and frequency of injuries.

### Statistical analysis

Statistical analysis was performed using R (version 4.1.2) and epitools (version 0.5–10.1) package. We examined the ESAW code counts of the economic activity sectors. Any dissimilarity between them was illustrated using classical multidimensional scaling (MDS), into two principal coordinates with Euclidean distances. Risk ratios (RR) were used to assess the associations between the individual ESAW codes and the economic activity sectors. Confidence intervals (CI) were approximated using Wald’s method.

### Ethics

The database material was analysed without personal data, preventing any identification of individuals. Data were protected in line with European Union General Data Protection Regulation (GDPR). As we did not contact the cases, neither patient consent nor approval was required from the ethics committee.

## Results

A total of 37,986 work-related head injuries occurred in 2010–2017. Commuting (*n* = 2669) and leisure-time (*n* = 2418) injuries and suspected or verified occupational diseases (*n* = 1) were excluded, resulting in a final number of 32,898 work-related injuries in the study. Most of the injured employees (69.6%) were male. All the participants were ≥ 14 years old. There were no major differences between the sex distribution of the different age groups.

**Fig. 1:**
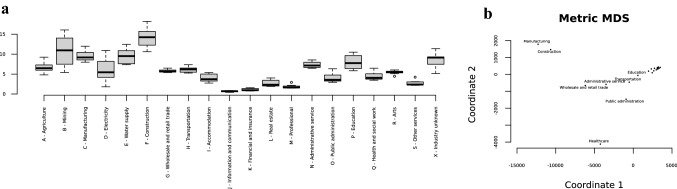
**a** Distribution of annual occupational head injuries relative to 1000 insurance years in different industry sectors in 2010–2017, Finland. x-axis: industry sectors, y-axis: injury density. Industry sectors according to Standard Industrial Classification (TOL) 2008, Statistics Finland, and Statistical Classification of Economic Activities in the European Community (NACE). **b** Multidimensional scaling of ESAW category counts into 2 dimensions in 20 different industry sectors according to occupational head injuries in 2010–2017, Finland. x-axis: coordinate 1 (MDS1) y-axis: coordinate 2 (MDS2). Industry sectors according to Standard Industrial Classification (TOL) 2008, Statistics Finland, and Statistical Classification of Economic Activities in the European Community (NACE). ESAW (European Statistics on Accidents At Work)

The box plots of the annual injury densities in 2010–2017 indicated highly varied risk profiles across the industries (Fig. 1). Some industries, such as construction, had consistently high injury incidence causing head traumas. In some other high-risk industries, such as mining, the actual densities varied to a greater extent annually. At the other end of the scale, some industries, such as information and communications, had consistently low head injury rates. Two minor industries, employers of households (TOL 2008 code T) and extraterritorial organisations (TOL code U), were excluded, as no head injuries occurred in most years, and any single event would be shown as an extreme outlier.

Multidimensional scaling of ESAW category counts into two dimensions illustrated the distances between the different industries (Fig. 1). The main concentrated group in the illustration consisted of most of the industries, indicating only small differences between them. Three industries (construction, manufacturing and health care) differed from this main group. Table 1 shows the characteristics of the work-related injuries and the part of the head injured in these 3 industry sectors, as well as in all 20 sectors. The incidence of injuries per 1000 insurance years was highest in construction (15.7%). The mean age of male and female employees was similar in all three sectors. There was a clear male majority in construction and manufacturing and a female majority in human health and social work activities.

**Table 1:** Work-related head injuries in 2010–2017 in three different main industry sectors, Finland

Parameter	All 20 sectors *n* (%) = 32,898 (100.0)	Construction *n* (%) = 5920 (18.0)	Manufacturing *n* (%) = 6530 (19.8)	Health^a^ *n* (%) = 3834 (11.7)
Incidence of injuries per 1000 insurance years	6.8	15.7	10.6	4.8
Sex (*n*, %)				
Male	22,895 (69.6)	5822 (98.3)	5929 (90.8)	722 (18.8)
Female	10,003 (30.4)	98 (1.7)	601 (9.2)	3112 (81.2)
Age (mean)				
All	38.5	37.2	39.6	40.2
Male	38.1	37.2	39.4	38.7
Female	39.5	34.6	41.2	40.5
Part of head injured^b^				
Head (Caput), brain and cranial nerves and vessel	6925 (21.0)	645 (10.9)	970 (14.9)	1110 (29.0)
Facial area	5931 (18.0)	604 (10.2)	761 (11.7)	1246 (32.5)
Eye(s)	16,311 (49.6)	4169 (70.4)	4324 (66.2)	971 (25.3)
Ear(s)	722 (2.2)	72 (1.2)	123 (1.9)	84 (2.2)
Teeth	1878 (5.7)	325 (5.5)	220 (3.4)	143 (3.7)
Head, multiple sites affected	376 (1.1)	28 (0.5)	33 (0.5)	154 (4.0)
Head, other parts not listed	755 (2.3)	77 (1.3)	99 (1.5)	126 (3.3)

Industry sectors according to Standard Industrial Classification (TOL) 2008, Statistics Finland, and Statistical Classification of Economic Activities in the European Community (NACE)

^a^Human health and social work activities

^b^Part of head injured according to ESAW (European Statistics on Accidents at Work)

On the basis of ESAW information derived from employers’ reports, the most commonly affected areas in work-related head injuries in our material were the eyes (49.6%), the brain and cranial nerves and vessels (21.0%), and the facial area (18.0%) (Table 1). The eyes were also the most often injured area of the head in construction (70.4%) and manufacturing (66.2%), whereas only 25.3% of the injuries in human health and social work activities involved the eyes. The facial area (32.5%), and the brain and cranial nerves and vessels (29.0%) were injured most often in human health and social work activities, in contrast to construction and manufacturing, in which these areas were not commonly affected.

The most common ‘specific physical activities’ (according to ESAW) connected to work-related injuries performed by the employees just before the injuries were ‘movement’, ‘handling of objects’ and ‘working with hand-held tools’ (Table 2). Compared with all other industries, ‘working with hand-held tools’ (RR 2.23, 95% CI 2.14–2.32) in construction and ‘operating machine’ (RR 3.32, 95% CI 3.01–3.66) and ‘working with hand-held tools’ (RR 1.99, 95% CI 1.91–2.07) in manufacturing were much more significant predictors of work-related head injuries. In contrast, these two activities were negatively associated with work-related head injuries in human health and social work activities. Unlike construction and manufacturing, ‘movement’ (RR 1.85, 95% CI 1.78–1.92), ‘presence’ (RR 2.65, 95% CI 2.40–2.91) and ‘other specific physical activities not listed’ (RR 2.80, 95% CI 2.30–3.40) were predictors of work-related head injuries in human health and social work activities.

**Table 2:** Specific physical activity connected to work-related head injuries in 2010–2017 in three different main industry sectors compared to other industry sectors, Finland

Specific physical activity^a^	*n*	RR (95% CI)
Operating machine (*n* = 1472)		
Construction	294	1.14 (1.00–1.29)
Manufacturing	664	3.32 (3.01–3.66)
Health^b^	19	0.10 (0.06–0.16)
Working with hand-held tools (*n* = 6906)		
Construction	2267	2.23 (2.14–2.32)
Manufacturing	2278	1.99 (1.91–2.07)
Health	86	0.10 (0.08–0.12)
Driving/being on board a means of transport or handling equipment (*n* = 603)		
Construction	71	0.61 (0.48–0.78)
Manufacturing	72	0.55 (0.43–0.70)
Health	16	0.21 (0.13–0.34)
Handling of objects (*n* = 9167)		
Construction	1492	0.89 (0.84–0.93)
Manufacturing	1690	0.91 (0.87–0.95)
Health	963	0.89 (0.84–0.94)
Carrying by hand (*n* = 1521)		
Construction	178	0.60 (0.52–0.70)
Manufacturing	206	0.63 (0.55–0.73)
Health	130	0.71 (0.59–0.85)
Movement (*n* = 9436)		
Construction	1011	0.55 (0.52–0.58)
Manufacturing	1105	0.54 (0.51–0.57)
Health	1852	1.85 (1.78–1.92)
Presence (*n* = 1956)		
Construction	209	0.53 (0.46–0.61)
Manufacturing	176	0.40 (0.34–0.47)
Health	506	2.65 (2.40–2.91)
Other specific physical activities not listed (*n* = 494)		
Construction	68	0.73 (0.56–0.94)
Manufacturing	62	0.58 (0.44–0.76)
Health	133	2.80 (2.30–3.40)
No information (*n* = 1343)		
Construction	330	1.88 (1.65–2.14)
Manufacturing	277	1.17 (1.01–1.34)
Health	129	0.83 (0.68–1.01)

Industry sectors according to Standard Industrial Classification (TOL) 2008, Statistics Finland, and Statistical Classification of Economic Activities in the European Community (NACE)

^a^Specific physical activity according to ESAW (European Statistics on Accidents at Work)

^b^Human health and social work activities

‘Deviation by overflow, overturn, leak, flow, vaporisation, emission’ was by far the most common abnormal event related to work-related injuries (Table 3). ‘Shock, fright, violence, aggression, threat and presence’ had an almost ninefold higher risk and ‘deviation due to electrical problems, explosion and fire’ had an over fourfold higher risk of work-related head injury in human health and social work activities than in other industry sectors. On the other hand, the same deviations were negatively associated with work-related injuries in construction and manufacturing.

**Table 3:** Deviation of norm performance connected to work-related head injuries in 2010–2017 in three different main industry sectors compared to other industry sectors, Finland

Deviation^a^	*n*	RR (95% CI)
Due to electrical problems, explosion, fire (*n* = 324)		
Construction	34	0.54 (0.38–0.77)
Manufacturing	49	0.73 (0.54–0.99)
Health^b^	117	4.37 (3.49–5.47)
By overflow, overturn, leak, flow, vaporisation, emission (*n* = 13,237)		
Construction	3492	1.72 (1.67–1.76)
Manufacturing	3773	1.69 (1.65–1.74)
Health	860	0.55 (0.52–0.58)
Breakage, bursting, splitting, slipping, fall, collapse of material agent (*n* = 5261)		
Construction	808	0.87 (0.81–0.94)
Manufacturing	848	0.82 (0.77–0.88)
Health	388	0.63 (0.58–0.70)
Loss of control of machine, means of transport or handling equipment, hand-held tool, object, animal (*n* = 2319)		
Construction	460	1.19 (1.08–1.31)
Manufacturing	440	1.00 (0.90–1.10)
Health	133	0.48 (0.41–0.57)
Slipping—stumbling and falling—fall of persons (*n* = 2203)		
Construction	245	0.60 (0.53–0.69)
Manufacturing	207	0.44 (0.38–0.51)
Health	221	0.89 (0.78–1.02)
Body movement without any physical stress (*n* = 5908)		
Construction	691	0.63 (0.59–0.68)
Manufacturing	1017	0.88 (0.83–0.94)
Health	724	1.11 (1.03–1.19)
Body movement under or with physical stress (*n* = 211)		
Construction	14	0.33 (0.19–0.57)
Manufacturing	19	0.41 (0.25–0.65)
Health	32	1.38 (0.95–2.01)
Shock, fright, violence, aggression, threat, presence (*n* = 2378)		
Construction	31	0.06 (0.04–0.09)
Manufacturing	10	0.02 (0.01–0.03)
Health	1249	8.92 (8.27–9.61)
Other deviations not listed above in this classification (*n* = 629)		
Construction	91	0.81 (0.65–1.01)
Manufacturing	99	0.79 (0.64–0.98)
Health	69	0.98 (0.76–1.26)
No information (*n* = 428)		
Construction	54	0.67 (0.51–0.90)
Manufacturing	68	0.78 (0.60–1.01)
Health	41	0.82 (0.60–1.13)

Industry sectors according to Standard Industrial Classification (TOL) 2008, Statistics Finland, and Statistical Classification of Economic Activities in the European Community (NACE)

^a^Deviation according to ESAW (European Statistics on Accidents at Work

^b^Human health and social work activities

Analysis of the ESAW ‘mode of injury’ (Table 4) revealed that the most common type was ‘struck by object in motion and collision with’, followed by ‘contact with hazardous substances—on/through skin or eyes’ and ‘horizontal or vertical impact with or against a stationary object’. In the human health and social work activities, the employees were injured significantly more often because of ‘contact with hazardous substances through nose, mouth or via inhalation’ (RR 13.48, 95% CI 10.29–17.66) and ‘bite, kick, etc.’ (RR 8.40 95% CI 7.79–9.07) than in the other industries. In contrast, ‘bite, kick, etc.’ were rare incidents in construction and manufacturing.

**Table 4:** Mode of injury connected to work-related head injuries in 2010–2017 in three different main industry sectors compared to other industry sectors, Finland

Mode of injury^a^	*n*	RR (95% CI)
Indirect contact with a welding arch, spark, lightning (passive) (*n* = 171)		
Construction	40	1.39 (0.98–1.98)
Manufacturing	69	2.73 (2.01–3.70)
Health^b^	5	0.23 (0.09–0.56)
Direct contact with electricity, receipt of electrical charge in the body (*n* = 21)		
Construction	9	3.42 (1.44–8.11)
Manufacturing	5	1.26 (0.46–3.44)
Health	1	0.38 (0.05–2.82)
Contact with naked flame or a hot or burning object or environment (*n *= 262)		
Construction	42	0.87 (0.62–1.21)
Manufacturing	53	1.02 (0.76–1.38)
Health	22	0.69 (0.45–1.07)
Contact with a cold or frozen object or environment (*n* = 10)		
Construction	0	na
Manufacturing	2	1.01 (0.21–4.75)
Health	1	0.84 (0.11–6.65)
Contact with hazardous substances—through nose, mouth via inhalation (*n* = 225)		
Construction	9	0.19 (0.10–0.37)
Manufacturing	20	0.39 (0.25–0.62)
Health	144	13.48 (10.29–17.66)
Contact with hazardous substances—on/through skin or eyes (*n* = 5374)		
Construction	1079	1.14 (1.08–1.22)
Manufacturing	1296	1.28 (1.21–1.36)
Health	630	1.01 (0.93–1.09)
Contact with hazardous substances—through the digestive system by swallowing or eating (n = 43)		
Construction	7	0.89 (0.39–1.99)
Manufacturing	5	0.53 (0.21–1.35)
Health	12	2.93 (1.51–5.71)
Other mode of contact with electrical voltage, temperature, hazardous substances injury (n = 131)		
Construction	21	0.87 (0.55–1.39)
Manufacturing	37	1.59 (1.09–2.32)
Health	9	0.56 (0.28–1.10)
Drowned, buried, enveloped (n = 17)		
Construction	6	2.49 (0.92–6.72)
Manufacturing	4	1.24 (0.41–3.81)
Health	0	0
Horizontal or vertical impact with or against a stationary object (the victim is in motion) (*n* = 5367)		
Construction	511	0.48 (0.44–0.52)
Manufacturing	687	0.60 (0.55–0.64)
Health	704	1.14 (1.07–1.22)
Struck by object in motion, collision with (*n* = 12,338)		
Construction	2813	1.35 (1.30–1.39)
Manufacturing	2788	1.18 (1.14–1.22)
Health	705	0.46 (0.43–0.49)
Contact with sharp, pointed, rough, coarse material agent (*n* = 5035)		
Construction	1167	1.37 (1.30–1.46)
Manufacturing	1314	1.43 (1.35–1.51)
Health	237	0.37 (0.33–0.42)
Trapped, crushed, etc. (*n* = 417)		
Construction	34	0.40 (0.29–0.57)
Manufacturing	72	0.84 (0.65–1.08)
Health	30	0.59 (0.41–0.85)
Physical or mental stress (*n* = 427)		
Construction	26	0.30 (0.20–0.44)
Manufacturing	56	0.61 (0.46–0.81)
Health	55	1.12 (0.85–1.48)
Bite, kick, etc. (animal or human) (*n* = 2214)		
Construction	28	0.06 (0.04–0.08)
Manufacturing	7	0.01 (0.01–0.03)
Health	1164	8.40 (7.79–9.07)
Other contacts (*n* = 499)		
Construction	89	0.99 (0.79–1.24)
Manufacturing	62	0.57 (0.44–0.75)
Health	78	1.40 (1.11–1.78)
No information (*n* = 347)		
Construction	39	0.58 (0.41–0.80)
Manufacturing	53	0.73 (0.54–0.97)
Health	37	0.90 (0.64–1.27)

Industry sectors according to Standard Industrial Classification (TOL) 2008, Statistics Finland, and Statistical Classification of Economic Activities in the European Community (NACE)

^a^According to ESAW (European Statistics on Accidents at Work

^b^Human health and social work activities

## Discussion

The aim of this study was to investigate the work-related risks and pathways that precede head injuries, which is essential for promoting industry-specific safety. We found considerable variation in the circumstances across industries, with the highest injury incidences being in construction. In our material, the most commonly affected area was the eyes. In addition, clearly more head injuries were caused by violence in human health and social work activities than in the other sectors.

The main strength of the current study was its extensive data on 32,898 work-related injuries, based on a national system of statutory insurance and reporting, in which the percentage of non-reported injuries is low. However, the setting is also a limitation of the study, particularly in terms of the accuracy of information recorded by the employer.

Comparison of occupational accident data is difficult due to the different data collection and recording practises in different countries. ESAW was launched to harmonise this data (EU 2013), but the coding reliability of ESAW variables varies, and some variables are difficult to understand and code without training (Jacinto et al. 2016). The ESAW ‘age’, ‘sex’ and ‘nationality’ variables have shown good coding reliability, whereas the variables concerning accidents, such as ‘deviation’, have shown ‘low to moderate’ reliability (Molinero-Ruiz et al. 2015). To improve the reliability in our setting, we combined all the ESAW variables (variables 11–19) and combined the injuries to different areas of the head into one variable (‘head injury’), as the employer, who is responsible for the accident report, is not usually educated in health care.

National differences in the accuracy of recording work-related injuries, and underreporting of these are a recognised challenge (Eurofound 2017; Jacinto and Aspinwall 2004). The level of reporting is higher in countries with insurance-based systems. In contrast, the reporting level can be as low as 30% (Jacinto and Aspinwall 2004) in countries with non-insurance-based systems. Detailed data on work-related injuries are available in the Nordic countries, including Finland (Jacinto and Aspinwall 2004), where worker’s compensation insurance, is in a primary position compared to other social security benefits, with significant financial compensation for the victim. In Finland, less than 10% of the employees are not covered, reasons being related to amount of salaries paid by employer, and in some cases neglect. Consequently, the current material is likely to reflect the overall national situation.

Some of the employees in our data were 14 years old, because of work during school holidays and short internships at workplaces as part of the school year. The oldest age group of the cases were 70–79 years, as some retired people still worked. Comparison of exact age and gender distribution with all Finnish employees is not possible due to national insurance practises and the European Union General Data Protection Regulation (GDPR).

The work-related injury densities and risk profiles of the different industries varied greatly in the current results. Construction had a consistently high density of injuries; whereas at the other end of the scale was information and communications, which had consistently low injury density. Mining also had a high injury density, but the size of the industry was small, with wide variation in injury density. The ESAW subcategory counts stood out in the three main industry sectors (human health and social work activities, construction, and manufacturing) from the others. We selected these sectors for further analyses, which revealed differences in a wide range of subclasses of the ESAW variables (specific physical activity, deviation of norm, and modes of injury).

Previous studies (Haslam et al. 2005; Jaafar et al. 2018; Ren et al. 2008) have presented different models to explain the pathways related to injuries at work; many of these being developed specifically for the construction industry in which the incidence of non-fatal injuries has been high (EU 2020). Several consecutive errors are usually behind an accident and the failure of safety factors (Ren et al. 2008). Moreover, both distal (e.g. organisation, society) and proximal (e.g. site condition, individual characteristics) factors are important when exploring the causes of work-related injuries (Khosravi et al. 2014).

Previous studies have shown that about half of eye traumas (34–54%) are work related (Cai and Zhang 2015; Sahraravand et al. 2017). In our study, the most common area of the head that was affected was the eyes (49.6%). Both in construction (70.4%) and manufacturing (66.2%), eye injuries were the leading form of head injury. Of these eye injuries, 83% occurred among men, which is similar to the results of a previous study by Martin-Prieto et al. (2020). Thus, attention to safety and eye protection is still required.

In general, the risk of work-related accidents in the construction industry is high (EU 2020). Consistent with a previous systematic review (Chang et al. 2015), we observed the highest incidence of head injuries to be in construction. Falls are the main cause of TBI in the construction industry (Brolin et al. 2021; Colantonio et al. 2009; Kim et al. 2006). In our material, which was not restricted to TBI, falls did not emerge in the context of the injuries, as the eyes were the most commonly affected area. In construction and manufacturing, current head injuries occurred due to the typical stages of the work in question, such as ‘working with hand-held tools’.

The circumstances behind the work-related head injuries in the human health and social work activities sector were different to those in all the other sectors. When examining the ‘deviation’ and ‘mode of injury’, behind the head injuries in the human health and social work activities, ‘shock, fright, violence, aggression, threat, presence’ and ‘bite, kick, etc.’ showed almost a ninefold higher risk of head injury compared to other industry sectors, suggesting violence being a major factor behind the head injuries in this industry. In addition, ‘Contact with hazardous substances—through the nose, mouth via inhalation’ showed an almost 14-fold risk of head injuries, which further emphasises the differences between industry sectors.

Workplace violence has received increasing attention in recent years. The European Working Conditions Survey (EWCS) (Eurofound 2017) revealed that 2% of workers in 35 European countries were exposed to workplace physical violence in the last 12 months, and health and social work employees reported the highest percentage (7%) (Eurofound 2013). Some TBI has been indicated in health care and social assistance (Shafi et al. 2019), and our study contributed by showing a clear excess of head injuries in the human health and social work sector. The threat of violence may be more common in occupations in which the work involves a great deal of external client contact, as is the case in health care (Eurofound 2013). The construction industry involves less interaction with people outside the organisation and the sites, which may contribute to its lower incidence of violence. A systematic review and meta-analysis by Liu et al. (2019) showed that workplace violence is high in the health care sector, especially among nurses and physicians. In our study, we did not separate employees by profession. A study by Torre et al. (2022) highlighted the difficulty to assess the prevalence of violence experienced by health care workers due to under-reportment or un-reportment of the violence. However, when investigating the more serious consequences of workplace violence, such as head injury, we can assume that most of the occupational accidents resulting from violence are reported. A previous study using a sample from an occupational injury database of the same Finnish insurance company as that in our study showed reoccurrence of violence-related accidents for every other subject (Pietilä et al. 2018). Therefore, analysing accidents can be important for preventing the probability of incidents.

## Conclusion

Data based on a national system of statutory insurance and reporting, including ESAW codes, enabled us to improve the description of the pathways of head injuries in various industry sectors. In construction and manufacturing, the injuries happened due to typical stages of work in question, such as ‘working with hand-held tools’. Health care emerged with the most distinctive violence and threat-related profiles due to the nature of the sector. Thus, when developing occupational safety to prevent head injuries, attention should be paid to the injury-specific circumstances, including sex distribution, and the nature of the work and the workplace. In the construction and manufacturing, occupational safety must still be improved by developing the safety of working tools and machines as well as by improving safety equipment. In social and health care, in addition to the work environment, attention must be paid to the employee’s ability to interpret situations and behaviour indicating a risk of violence.

## Data Availability

As data are derived from statutory workers’ compensation insurance, it cannot be made available due to restrictions related to GDPR.
